# Comparative Diagnosis of Serum IgG1 and Coproantigen ELISA for Fasciolosis Detection of Goats in Mexico

**DOI:** 10.1155/2016/3860928

**Published:** 2016-08-01

**Authors:** Abel Villa-Mancera, Pedro Molina-Mendoza, Karina Hernández-Guzmán, Jaime Olivares-Pérez, Jorge Sarracent-Pérez, José Zumaquero-Ríos

**Affiliations:** ^1^Laboratorio de Biotecnología Agropecuaria y Biología Molecular, Facultad de Medicina Veterinaria y Zootecnia, Benemérita Universidad Autónoma de Puebla, 75482 Tecamachalco, PUE, Mexico; ^2^Unidad Académica de Medicina Veterinaria y Zootecnia, Universidad Autónoma de Guerrero, 40660 Ciudad Altamirano, GRO, Mexico; ^3^Laboratorio de Hibridomas, Instituto de Medicina Tropical Pedro Kourí, La Habana, Cuba; ^4^Laboratorio de Parásitos y Vectores, Escuela de Biología, Benemérita Universidad Autónoma de Puebla, 72000 Puebla, PUE, Mexico

## Abstract

The objective of present study was to determine the prevalence of natural caprine fasciolosis in the Mixteca region of Mexico using coproantigen and serum IgG1 ELISA tests for comparative purposes. A total of 1070 serum and faecal samples were analyzed for IgG1 antibodies and coproantigens, using ELISA with E/S products as antigen and a monoclonal antibody-based sandwich ELISA. Prevalence of 73.46% was found using the serological ELISA and a percentage of 77.20 was found for coproantigen ELISA. The diagnostic sensitivity and specificity for serum ELISA were 86.7% and 96.4%, and for the coproantigen ELISA they were 93.1% and 97.8%, respectively. The seropositive samples were further categorized as low, medium, or high positivity. Results show a great proportion of low and medium positive goats when the serum ELISA test was used. Correlation coefficients between coproantigens and seropositivity were statistically significant (*P* < 0.01) for low seropositivity (*r* = 0.93) and medium seropositivity (*r* = 0.84). The accuracy of faecal antigen ELISA was higher compared to indirect ELISA serological test. Two ELISAs were shown to be useful for demonstrating the current status of* F. hepatica* infection in the endemic areas and can be employed in studies on epidemiology as well as anthelmintics treatment for preventing economic loss and the risk of transmission to humans.

## 1. Introduction

Fasciolosis is a foodborne zoonotic disease that affects grazing animals and humans.* Fasciola hepatica* causes global economic losses to the agriculture, estimated at over three billion US dollars every year [1]. At least 90 million people are at risk of infection and between 2.4 and 17 million individuals are currently infected [2]. The pathogenic trematode is widespread, causing infections in Bolivia, Peru, Ecuador, Egypt, and Iran [3–5].

Although caprine infection is lower than ovine or bovine fasciolosis, goats are extremely sensitive and susceptible to both natural and experimental infections [6, 7].* F. hepatica* has been also detected in temperate cooler areas of high lands of tropical and subtropical regions [8]. Mexico possesses 8.6 million goats [9] located in areas with lowest human development index, with high potential to improve economy. High* Fasciola* prevalence in goats has been reported in the northwest Mexico using the indirect ELISA and sedimentation tests (43.0% and 24.5% [10]).

The economic importance of fasciolosis is attributed to the loss of livers in abattoirs [11], reduced feed efficiency and milk production, delayed animal growth, reproductive insufficiency, losses due to animal morbidity and mortality, and cost of treatment [12–15].

Rapid, early, and accurate diagnosis of the infection is key to studying the epidemiology of fascioliasis and the surveillance and control of this disease. Antemortem tools for the detection of fasciolosis, ranging from copromicroscopic techniques to immunodiagnostics and molecular diagnostics, have been utilised [16, 17]. Several studies had been performed on parasite antigens in faeces (coproantigens) and immunodiagnosis of* F. hepatica* infection in livestock (particularly sheep and cattle), while fewer studies had been done on diagnosis of liver fluke disease in goats [15, 17]. The aim of the present study was to compare the performances of monoclonal antibody-based sandwich ELISA in faeces (coproantigens) and serum IgG1 ELISA test for the diagnosis of* Fasciola hepatica*. This study was performed with a panel of samples from goats under the transhumant system in the Mixteca region of Mexico.

## 2. Methods

### 2.1. Study Area, Goat Breeds, and Sample Collection

**Figure 1:**
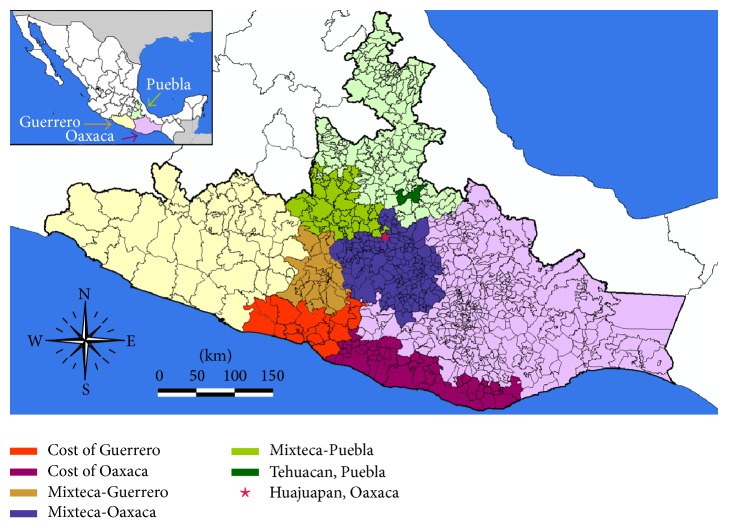
Map of the Mixteca region of Mexico and places mentioned in the text.

The Mixteca region includes portions of three states of southern Mexico: (1) the western part of Oaxaca, (2) the eastern border of Guerrero, and (3) southern Puebla (Figure 1). It is divided into three subregions. The Mixteca Alta is located between the two other subregions; it is a high, temperate, and cool region of mountains and small valley. The Mixteca Baja refers to the arid regions at lower elevations of northern Mixteca Alta and extends north into Puebla. The Mixteca de la Costa covers the Pacific Coast to the northern mountains of Mixteca Alta region. The Mixteca region of Mexico has a great diversity of tree and shrub species which becomes important forage resources consumed by transhumance goats. Map of the Mixteca region of Mexico was generated using ArcGIS 10.1 (ESRI; Redlands, CA, USA).

The Creole goat is a dominant breed used for meat production in the Mixteca region. Other breeds such as Boer, Nubia, Alpina, and their crosses have demonstrated considerable genetic merit to improve growth traits, mainly under extensive management conditions.

Producers purchase the goats grazing on the coast of Guerrero and Oaxaca and transported to grazing land in the Mixteca (May to November); then, the fattening goats were sent to Tehuacan, Puebla, and Huajuapan, Oaxaca, and sampled between October and November (autumn 2014). Blood and faecal samples from 1070 animals were collected from Mixteca-Guerrero and Puebla (*n* = 698) and Mixteca-Oaxaca (*n* = 372) and transported to the laboratory of Agricultural Biotechnology and Molecular Biology. Five grams of each faecal sample was processed individually using the sedimentation technique. Faecal eluates were prepared by adding 4 mL of phosphate-buffered saline/0.05% (v/v) Tween 20 (PBS-T) to 1 g of fresh faeces in a centrifuge tube. The mixture was homogenized and then centrifuged at 900 ×g for 5 min after which the supernatants were collected. Blood samples were centrifuged (3,500 ×g) for 10 min and supernatant eluates and serum samples were stored at −80°C until use.

Castrated male and female goats were fattened under an extensive production system, usually with grazing transhumance. Goats graze at high altitude during the rainy season and move to low altitude in cold weather, searching for better availability of fodder and weather conditions. Large herds graze on rented communal lands.

### 2.2. Adult* F. hepatica* Excretion/Secretion Products (E/S)

*Fasciola hepatica* of caprine origin were acquired in our previous work [18]. Adult fluke E/S products were obtained by incubating mature parasites for 16 h at 37°C in RPMI-1640 supplemented with penicillin (100 IU/mL) and streptomycin (100 *μ*g/mL). The supernatant was removed and centrifuged at 14000 ×g for 30 min at 4°C. E/S products were collected and concentrated in 10 kDa cut-off membrane Amicon Ultra-15 centrifugal filter tubes (Millipore, USA) and stored in aliquots at −80°C.

### 2.3. Positive and Negative Enzyme Linked Immunosorbent Assay (ELISA) Controls

Four goats below 1 year of age receiving different doses of* F. hepatica* (250 and 300 metacercariae from infected* Lymnaea cubensis* host snail culture) acted as positive control. The presence of parasite eggs in faecal samples was performed using the sedimentation technique. Negative control was collected from parasite naïve goats (serum and faecal samples). All control serum samples were analyzed using an ELISA kit (DRG International Inc., USA) following the manufacturer's specifications to detect antibodies against E/S products of* F. hepatica*.

### 2.4. Detection of Anti-*F. hepatica* IgG1 Antibodies in Serum by ELISA

The ELISA was optimized by checkerboard titration to determine the optimal concentration of antigen, serum, and conjugate dilutions. ELISA plates (Costar, Corning, NY, USA) were coated with 10 *μ*g/mL of E/S products in 100 *μ*L of PBS and incubated overnight at 4°C. After four washes with PBS-T, nonspecific binding sites were blocked with 200 *μ*L containing 1% bovine serum albumin (BSA) for 1 h at 37°C. Negative and positive controls and serum samples were used at a dilution of 1 : 400 in PBS and incubated at 37°C for 1 h. Microplates were washed four times with PBS-T and incubated with biotinylated sheep anti-bovine IgG1 isotype (1 : 10000, Abcam, USA; this antibody reacts with goats) for 1 h at room temperature in PBS-BSA 1%. Plates were washed five times, each time with PBS-T. The detection of isotype IgG1 antibody was carried out using HRP-streptavidin conjugate (1 : 5000, Invitrogen, USA). Following incubation, plates were washed five times with PBS-T. Lastly, the reaction was developed by the addition of 100 *μ*L per well of TMB (Sigma, USA). The enzyme-substrate reaction was stopped with 50 *μ*L of 4N H_2_SO_4_. Absorbance was measured at 450 nm in ELISA reader (BioTek ELx800). All serum samples and negative and positive controls were tested in triplicate on each plate. The antibody levels were expressed as an optical density index (ODI) using the following formula: [(OD test sample)/(OD positive control) ] × 100.

### 2.5. Detection of Coproantigen by Monoclonal Antibody- (MoAb-) Based Sandwich ELISA

The optimal concentration of ES-78 MoAb [19] for coating plates for ELISA was determined by checkerboard titration using a positive-control and negative-control stool samples. For each step, 100 *μ*L/well was added unless otherwise stated. The plates were sensitized overnight at 4°C with MoAb (10 *μ*g/mL in PBS). After four washes with PBS-T, unbound sites were blocked with 200 *μ*L of 1% BSA in PBS-T for 1 h at 37°C. Undiluted faecal eluates were added and the plates were incubated at 37°C for 1 h. After thorough washing as described above, HRP-conjugated rabbit anti-*F. hepatica* E/S products IgG (dilution 1 : 2500) in PBS-BSA 1% was added. The microplates were incubated for 1 h at 37°C and washed with PBS-T. Colour reaction was developed by the addition of TMB substrate (Sigma, USA) and read at 450 nm.

### 2.6. Statistical Analysis

The diagnostic sensitivity, specificity, positive predictive values (PPV), and negative predictive values (NPV) were calculated [20]. Microscopy serves as the gold standard for true positive and true negative. Pearson correlation coefficients were used to assess the relationship among serum IgG1 (low, medium, and high positivity) and E/S antigens in faeces. *P* value greater than 0.05 was considered not significant and *P* value less than 0.01 was considered highly significant. Statistical analysis was done using IBM SPSS 20 software package (SPSS Inc., Chicago, USA) for Windows.

## 3. Results

### 3.1. Sensitivity, Specificity, and Cut-Off Value of the ELISA

The optimum diagnostic sensitivity, specificity, and cut-off value were calculated using either sera or faeces tests (Table 1). The cut-off value was the 25 percent positivity (PP) for serum ELISA test; goats with a PP less than 25 were considered to be negative. Seropositivity was divided into 3 categories: low (25 ≤ PP ≤ 50), medium (50 ≤ PP ≤ 100), and high (PP ≥ 100) positivity. For the coproantigen ELISA OD_450_ cut-off value for a positive result was defined at 0.5. Overall, the highest sensitivity and specificity were observed for the faecal antigen ELISA (93.1% and 97.8%, resp.), followed by the serological ELISA (86.7% and 96.4%, resp.). Likewise, the diagnostic accuracy of the assay was 95.1% and 91.6%. The serum and coproantigen ELISA showed high positive predictive values (proportion of goats that have fasciolosis with positive test results) of 98.5% and 99.3%, with negative predictive values (proportion of goats that have fasciolosis with negative test results) of 52.6% and 51.2%, respectively.

### 3.2. Prevalence of* F. hepatica* Infection

Coprological examination revealed prevalence of* Fasciola hepatica* in the Mixteca-Guerrero and Puebla of 59.45% (415/698) and in Mixteca of Oaxaca of 49.19% (183/372) (Table 2). The overall prevalence of* F. hepatica *infection in the Mixteca region, measured by serum antibody and coproantigen, was 73.46% (786/1070) and 77.20% (826/1070), respectively. The prevalence detected by coproantigens analysis was consistently higher than that detected by the serological test. The prevalence of* F. hepatica* infection performed on serum samples by ELISA, categorized as low, medium, or high positive, respectively, was 30.00%, 41.12%, and 2.34%. The highest seroprevalence for medium positivity was detected in 440 of 1070 samples; whereas the lowest prevalence for high positivity was observed in 25 goats. The correlation coefficients between serum IgG1 prevalence and E/S antigens in faeces are shown in Table 3. Coproantigen ELISA was significantly correlated with low and medium positivity, though *r* ^2^ values were particularly high (Pearson's correlation coefficient *r* = 0.93, *r* ^2^ = 0.86, and *P* < 0.01 and *r* = 0.84, *r* ^2^ = 0.70, and *P* < 0.01, resp.) but not significantly correlated with high positivity (*r* = −0.49; *P* = 0.32). Besides, the correlation among low positivity and medium positivity was higher and statistically significant (*r* ^2^ = 0.89; *P* < 0.01). A negative correlation was observed between high positivity and low positivity (*r* = −0.42; *P* = 0.40) and medium positivity (*r* = −0.60; *P* = 0.22).

## 4. Discussion

Traditional parasitological methods for diagnosis of* Fasciola* infections are usually based on the detection of parasite eggs in faeces, but trematode egg shedding is intermittent and irregular and does not detect immature stages of parasite. Copromicroscopy is labour-intensive and relatively unreliable. Detection of coproantigens by sandwich ELISA is a rapid, easy, and sensitive test compared to the liver fluke eggs in faeces [21]. Serological diagnosis by ELISA is the most sensitive and inexpensive technique for fascioliasis, detecting the infections earlier [22, 23].

In this study, a MoAb-based sandwich ELISA was employed for detection of circulating* F. hepatica* E/S products (nonglycosylated antigen: 14, 24, 26, and 51 kDa) in stool samples and anti-*F. hepatica* IgG1 antibodies in serum by indirect ELISA. Natural infection with* F. hepatica* metacercariae has been shown to produce high levels of IgG1 antibodies and virtually no IgG2, thereby eliciting a nonprotective Th2 cell response [24]. Furthermore, the sensitivity and specificity of ES-78 MoAb-based ELISA in faeces were 93.1% and 97.8%, while in serum ELISA they were 86.7% and 96.4%, respectively. Coproantigens were detected from 4 weeks after infection (wpi) in sheep using a MoAb-based sandwich ELISA [25]. E/S antigens in faeces of experimentally infected sheep could be detected from 4 wpi, with sensitivity of 93.3% [26]. Fluke E/S products recognized by antibodies in sera of infected goats can be detected from 2 wpi [27, 28]. The sensitivity and specificity values reported in the literature to detect anti-*F. hepatica* serum antibodies in sheep ranged from 68.2 to 100% and from 95 to 100%, respectively [29, 30]. In this trial, the diagnostic accuracy of ES-78 MoAb-based sandwich ELISA in stool was superior to serum samples (95.1% versus 91.6%); this could be due to the fact that coproantigens are less affected by immune complex formation [31].

The present study is the first report in the Mixteca region of Mexico in which indirect ELISA for the detection of serum antibodies was used to determine the prevalence of infection compared to a coproantigen test. The prevalence of infection was recorded to be higher for coproantigen ELISA (77.20%) than indirect ELISA serological test (73.46%). Additionally, 3.74% coproantigen-positive goats were found to be negative to serum IgG1 ELISA, which may indicate that the goat's immune system did not respond adequately to the antigenic stimulus from migrating or mature flukes [8]. Prevalence of 43.0% was detected using indirect ELISA in goats from a semidesert area in the northwest of Mexico [10]. In Galega goats from northwestern Spain, seroprevalence of 22.7% was found using capture ELISA (MM3 antigen comprised cathepsins L1 and L2 and a Kunitz-like protein) [32]. The prevalence of fasciolosis in Pakistan has been reported using a commercially available indirect ELISA kit (DRG, Germany) in goats and ranged between 4.08% and 49.36% [8, 33]. Over the past decade, the prevalence of* F. hepatica* infection has risen in part due to climate change, increased animal movement, and changing farming practices [34]. Other causes include age, sex, breed, husbandry, and protocols used for the treatment of fasciolosis [5]. The actual number of animal and human infections is likely to be much higher due to its asymptomatic nature, the limited availability of diagnostic tools, and the lack of systematic or coordinated reporting of infections, especially in undeveloped countries [35].

The anti-*F. hepatica* IgG1 ELISA result expressed as a positivity value (negative, low, medium, or high) estimates the concentration of antibodies in serum sample; it is a product of the prevalence and the intensity of infection in each infected goat. The results are in agreement with [36]. In the present study, the statistical analysis showed that the correlation between coproantigen and low seropositivity is very high (0.93). The highly significant positive correlation between coproantigens decreases as seropositivity increases. The presence of coproantigen indicates* F. hepatica* infection, while serology would indicate recent exposure. A significant correlation has been found among the presence of coproantigens, egg output and intensity of infection in sheep [25, 26], and cattle [37, 38].

Seasonally abundant parasitic infections have a profound influence on the migration patterns of transhumant groups (distance covered, number of movements, duration of stays in each location, and the direction of movement when the season changes), restricting their time in favourable grazing but unfavourable disease areas [39, 40]. The infection is present in areas with climatic and soil features for the establishment of the intermediate host snail.* F. hepatica* is transmitted mainly by snails of the Family Lymnaeidae which are frequent in the country [41].

The goats were bought from the coast of Guerrero and Oaxaca, mixed for a long grazing period, and may then acquire parasites from several farms. Livestock interact with wildlife on the grazing pasture and at drinking water points for the larger part of the year; fascioliosis affects a wide range of mammals [42]. Transhumance of goats has strongly and rapidly declined in our study area in the last few years and tended to prefer indoor confinement. Today, rural population is declining in Mexico due to migration to the United States and even those who remain in the goat meat sector are reluctant to practice transhumance.

## 5. Conclusion 

These data showed that, with the use of ELISA based on E/S products as antigen and a monoclonal antibody, caprine fascioliasis can be diagnosed (sensitivity ≥ 86.7% and specificity ≥ 96.4%) for arresting its negative impact on growth and productivity, preventing economic loss and the risk of transmission to humans. Further studies focusing on identifying the environmental factors and grazing management will be necessary for the establishment of an efficient control strategy of* F. hepatica*.

## Figures and Tables

**Table 1:** Evaluation of diagnostic values for detection of serum anti-*F. hepatica* IgG1 and E/S antigens in faeces.

	Sensitivity (%)	Specificity (%)	Positive predictive values (%)	Negative predictive values (%)
Coproscopy	75.2	98.3	98.2	56.5
Serum ELISA	86.7	96.4	98.5	52.6
Coproantigen ELISA	93.1	97.8	99.3	51.2

**Table 2:** Prevalence of *F. hepatica* infection in goats from Mixteca region of Mexico by serum and coproantigen ELISA (*n* = 1070).

	Number of goats samples	Prevalence (%)
*Coproscopy*		
Mixteca-Guerrero and Puebla	415	59.45
Mixteca-Oaxaca	183	49.19
*Seroprevalence*		
Negative, PP < 25	284	26.54
Low positivity, 25 ≤ PP ≤ 50	321	30.00
Medium positivity, 50 ≤ PP ≤ 100	440	41.12
High positivity PP ≥ 100	25	2.34
Seropositivity (95% CI)	786	73.46 (49.59–52.21)
*Coproantigen (95% CI)*	826	77.20 (1.05–1.15)

CI, confidence interval; PP, percent positivity.

**Table 3:** Pearson correlation coefficients between coproantigen and anti-*F. hepatica* IgG1 antibodies in goats (low, medium, and high positivity).

	Coproantigen	Seropositivity		
		Low	Medium	High
*Coproantigen*	—			
Low positivity	0.93	—		
Medium positivity	0.84	0.94	—	
High positivity	−0.49	−0.42	−0.60	—
